# Work

**Published:** 1891-05

**Authors:** J. S. Wight


### WORK.[1]

BY PROFESSOR J. S. WIGHT, M.D.

When one of your members requested me to say something to the Odontological Society, I came to the conclusion, after considerable thought, that there was one thing with which we were all familiar,—I mean *work.* On this subject we can all meet with a common purpose; about it we can talk with a common interest; from its consideration we can reap a common advantage.

Of all work under the sun, there is none so noble and just as the work that we do for others. A lazy man will sometimes work for himself; he is like a man in the swim, as they say, he swims or drowns; or he works lest he perish. He is like the beggar who importuned the comedian for alms; and when the alms were not given, and he remarked that we must all live, the comedian told him that there was not the least necessity for it in the world. But a truly noble man will work for others, for he knows that he not

---

[1] Read before the Odontological Society of New York, February 17, 1891.

only helps others, but in this way he truly helps himself. Do you know that I was asking myself what kind of head-work is the best? And then I thought, that is best which most clearly and logically leads to the betterment of those around us. It does not consist in doing this thing or that thing, merely, but in doing the thing in such a way as to make it the most just for others. That work is best which is built upon what is just and right; from no other source can good head-work come, even in extracting teeth, or in sawing off bones. You may make your work ever so beautiful, but, if it is not just, if it is not right, it is not good. You say, the hand-work has been forgotten! No, I have not forgotten it; nor have I forgotten what a wonderful thing the hand is: how it labors for bread, for adornment, for science, for art, for fame; how it feeds the hungry; how it writes the story of our deeds, good and evil; how it executes the law; how it preaches the gospel and cures the sick; how it preserves teeth; how it works in every field of human endeavor under the sun, in all time and in all places. Neither do I forget how our art has been named after the hand, and that we are called surgeons, and that you are a part of surgery; and then I remember that neither you nor I have a handicraft,—we belong to a profession. Bear with me a little while, and I will tell you a few things that you ought to know, and which will draw the line between a handicraft and a profession.

Do you suppose that you work for yourselves? Does anybody suppose that you do? Does anybody believe that you alone of men are independent of others? Truly, as we labor at all, we labor for others. I cure their bones,—broken, to be sure; you cure their teeth in their autumnal decay, or give them new ones when the old are lost. Do you do it for yourselves? Whatever you do, you do it for another. You say, we do it for a price. Ah, then it is the pay you work for! We belong to a liberal profession. And I, once for all, dignify you with the title of special surgeons,—a part of our liberal profession. How do you like this designation? Do you like to be ranked among that ill-used race of men, among whom the liberality of Heaven brings the rewards of unselfish deeds down to the laps of thankful men? Now, in the name of that liberality which puts a crown upon the head of altruism, which is the patent of noble work, and which springs from the human heart, I plead with you, as I would with all men who labor, —I say it over and over again,—I plead with you to put your work first, to put it above all other things in this world. Put the best thought in it you can, for I can see no other way in which you can educate the hand to perform its highest duty than by making it ever and always responsive to execute those motions which arise from the best appointed and best cultivated head. The hand should be the facile instrument, quickly, precisely, and pleasantly, to execute and put into shape the ideas of a worker who places his work first. In the revelations of each new day, let the thought that we are doing our best be our inspiration. As I plead for the nobility of work, and as I magnify your profession and mine, I seem to hear some one say " *Tell us about the fee.*" Yes, I will tell you the laborer is worthy of his hire. How unjust it would be to deprive him of it, even though he till the fruitful soil, or sail the restless sea, or grind the nourishing corn, or bind the broken bone, or extract the painful tooth, or do whatever else is needful doing under the sun! But once more I tell you, and to you and all of us it may ever be told, that no man can make his hand-work of that high order which meets the approval of head and heart of the just and the true who first seeks his fee. His heart is in his fee, and his work is outside of his heart, and how could he put the best blood of his heart in his work? How could he put the best thought of his brain in his work, when he is seeking his fee? The best that we can do, the best work that any man can do, is wrought by the hand that is animated by a clear and liberal head, and that can rest with honor upon its owner's breast.

On a cold day in December I went to see a poor woman dying of cancer (I often stand with horror and amazement in the presence of this dread disease), my fee for which came down from heaven with the falling snow. I could not look upon that scene forever, so I started for home, facing the bitter wind and wishing that something could curtain the picture I had left. That something came soon enough, for I got into a horse-car, and in a little time there came in a weary laborer, with scant clothing which was patched and soiled, and in his eyes could be seen a misery and a hopelessness that seemed to be almost eternal and without remedy. He removed the tattered mittens from his horny hands, worn and roughened with work, and then I asked myself, "Does the man work for his fee first or last?" I noticed that the fingers of his left hand were for the most part gone,—worked off, perhaps, by machinery, or falling timbers, or stones. Often have I preserved such a hand for the laboring man, often without money and without price; and often I may do it again. As the conductor took the fare,—the road-fee,—a thought came into my mind,—"Why should not the road-company furnish passes to such men, who have but one hand, and who can therefore do only one-half hand-work, who work to support their wives and children?" Ah! no passes for such men as these, who do the dirty work, whose hands are rough, hard, and earnest,—these are the hands that built the road for others to ride on. I see the little books in the hands of honorable gentlemen who live without work, and who have contrived by some cunning of their own to make the rough hand-workers build roads for them to ride upon. Such men make others who have only one hand dig, drudge, and toil, in order that they may live by an inane kind of head-work. Then I put a fare into the hand of the humane conductor, who was also a hand-worker, and bade him give it to the one-handed workman, with the injunction not to tell him who did it. Now, let me say to you, that the kind of work that laboring-man does is without fee; it is real work; it is work built on everlasting law; it is built on principles as old, as eternal as the order of the universe.

Did it ever occur to you that if people did not get sick nor injured, they would not need doctors? And if teeth did not decay nor ache, they would not need dentists? I have sometimes thought, and particularly when I have had difficult cases, that if I had made, by evolution or in any other way, the individuals of our race, I would have so arranged the matter as to render them incapable of being sick, or so that no accident could harm them. But then there would be no broken bones for us to mend; nor would there be any filling or extracting of teeth; none of this great and fine work that you and the rest of us do. Then what would become of the money that is transformed into fees? Then what would become of you and me? Why, our bones would always be sound and whole. Our teeth would ever take the place of millstones and grind our wheat for us, and so prolong our lives. But do you not think that we could do some other hand-work quite as well as the one kind that we now do? For one moment think of the prospect of having all this skill engaged in the field of productive labor: I mean the skill that you and I possess. In a few years, how it would enrich the world, by leaving in the general aggregate all that is now abstracted, and then adding as much more to it. But you and I will have to keep on earning our fee, only we must see to it that our work is done for the sake of the work, and not for the fee.

You and I go down to the shore and save men, women, and children, for the sake of the fee; and for the same thing we take perishing mortality from the smoke, the flames, and the fire. It is an heroic, a noble, and a manly work, worthy of praise, worthy of imitation, worthy of remembrance. Yet, think of it! we live and get our living because others suffer pain; because they make mistakes; because of their errors; because of their follies and crimes; because of the calamities of the miserable; because of the misfortunes of the innocent and the unhappy. Think of all the human suffering that is summed up in these few words! Remember that our fee is for the attempt to relieve as much of this as we can. It is almost always an attempt that may or may not succeed. How little there is inside the boundary-line that limits our work,—they only know who do their head-work justly, rightly, wisely. All beyond this line is uncertain or impossible. Once I heard a good man say, "My work, when justly, rightly, and wisely done, doing all that I can do, and leaving undone all that I cannot do, is so pleasant that I would be glad to do it all, both night and day, in storm and in fair weather, if I could have food and raiment and shelter given me by some unseen hand, a *quid-pro-quo* kind of hand-work, for it is such a troublesome thing for me to get my fees." Like all true and noble things, such as are well done, and such as come from the heart that is in order with eternal justice, the work of this good doctor is immortal,—and his very dust, as it diffuses itself, will be grander and better because he was clothed in it when he wrought among men.

Let me ask you to think of two other things with which you and I are entirely familiar. They are so familiar that we give them little thought,—and that is one of the reasons why I ask you to think of them now. You know we invent little inventions, we make little improvements. You and I do this and it is an admirable thing to do; we get praise for doing it; it is the fruition of head-work, the flower of hand-work. Our names appear in the papers and we sometimes become famous, which is a very pleasant thing and a thing not to be rejected. What are these inventions that we invent? What are these improvements that we make? It would not be acceptable to my present purpose to give you a description of one and all of them now. You are all familiar with such things, and I do not wish to go over them and take up your time. It is more to the point for me to say that they are invented and made to facilitate,—remember that I say now and ever,—to facilitate *our* work. These inventions and improvements enable us to bring imperilled men, women, and children from the vexing and troubling sea that always moans without pity, and place them safely upon the solid land where they can defy the tempest. These inventions and improvements enable you and me to take from the smoke and flame and fire of danger and disaster the lives of those for whom we work. Just think of it; not for a long time, but long enough to get the idea into the cells of the brain. You certainly will not be offended if I tell you the truth? I have too much respect for your sense and your common sense to anticipate such a result as that. Let me say to you that this is one of the times when the truth must be spoken,—and spoken without reserve. Well, you have been getting a fee for your work all along; then you make an invention or an improvement, and with one or both of them you can do work quicker, easier, and better than you could before. But now you are not content with the fee you have been getting; you charge a greater fee for the same work; you know you ought not to do that, yet you do it all the same, as if you deserved it. But that is not all you do, for your ideas have expanded; you charge also for the invention and the improvement, and, what is more, you raise your fee again, because you have done your hand-work with this new piece of head-work,—this improvement on an invention or an improvement of some one else or even of your own,—so that work, expectations, and price all advance together. In this strange world of ours there is nothing stranger than this enigma, than this riddle of the fee-god.

Do you know, my friends, I am here in the most friendly way, and I know that the relation is reciprocal, that the members of my profession,—and I am trying to make you believe that you are a special part of it,—do you know that the members of my profession look upon your patents with concern and amazement? A patent is something that is open, so that everybody can see it; the patent opens the invention, the discovery, the improvement, to the world. Somebody finds out something by accident or by design, and then asks everybody to come and see it. He opens his invention, he shows everybody what it is; and everybody wants it. But this little invention, this patent thing, has a proviso attached to it. The government or the sovereign has seen it, and has given a grant to the inventor; and this grant contains the little proviso, which gives the inventor a monopoly: then he has a right to make and sell his invention at a profit, and he has also the right to put the profit into his own pocket. The patent and the right go together; and the theory is that the man has a right to his patent; that he has a patent-right to his invention; for is not his invention the product of the working of his own brain? What, it is said, could be plainer than this doctrine? Is not the laborer worthy of his hire? Is he not entitled to his wages? Are not his wages paid for his work? And is not his invention his work? Let me state the case in this way: You work; you invent an instrument; you get it patented; you acquire a right to make and use it; it is all yours, for you invented it; and now you ask everybody to pay you for using your invention. And then I seem to hear one say, I am like the author who writes a book; I am like the artist who paints a picture; one gets a copyright for his book-work; the other gets a copyright for his picture-work. Are you an artist? Are you an author? No; you are neither. You are a dentist. We will give the author and the artist their copyrights, whether they deserve them or not; that is, we will be liberal.

Now, there is another thing that I want you to think about,—and it is really a very important thing indeed. Think about it, after you have admitted that what I have said of the other side of the question is fair and just. I have not, however, repeated all the reasons and arguments in favor of the property-rights vested in inventions; but I have stated enough to show that there is such a thing as deserving a patent-right as well as a copyright. Once more: the other thing that I want you to think about is: The attitude of the Fathers of Medicine to this question of fee, as well as this question of invention. It is a strange fact of history that the Fathers of Medicine, at least in the earliest times, did not ask for, did not exact any fees. They did their work in the best manner they knew how, and then, if any one was grateful for the service rendered, and gave a fee in recognition of the fact, they took it with thanks, not in any other sense than because they deserved it. As you know, that has changed, and we now make regular charges; we send our bills, and sometimes we collect them.

Now, as to the question of patents, what was the attitude of those into whose labors we have entered? The grants of patent-rights; the making of money out of inventions; the keeping of valuable knowledge from the profession; the extortion of gain from the people,—they, whose footsteps we follow, never could be brought to see that such things were right; they never recorded any belief in their justice; they have never admitted that such things were wise; nor did they ever put any of them in practice. In fact, they did not apply the principle of hire and wages to matters of this kind; they ever held their high office as something too sacred to be levelled to the plane of patent-rights; they were governed by principles and precepts that had a deeper meaning, a wider application, a more desirable fruition. The practice involved in this matter may be illustrated by the proceedings of our societies of medicine and surgery,—and we believe the time is now at hand when we can include the Odontological Society in the same category. It appears—and it was and is an admirable thing—that in times present, as well as in times past, these gentlemen—this ill-used race of doctors,—I was going to say surgeons—came together and exchanged views on professional questions, and gave freely to each other of their experience and knowledge, much as you do in some other respects. These men were and are liberal, not only to each other, but also to the public. This liberality does not stop with medicine and bone-setting, but it extends to and includes the little inventions and the little improvements; they too are always freely given to others to have and use; they are not patented; for such a thing is derogatory to the dignity of a physician; it takes from the lustre of that work whose purpose is to cure and heal the sick and the injured. The inventions and the improvements of this curing and this healing art were and are cast upon the waters, and they not unfrequently return to us after many days, like that bread which was given to the poor and the needy. The Fathers of Medicine thought it wrong and unprofessional to have exclusive rights in the practice of their science and their art. We think the same, and so we practise. These principles, this practice, and this high ideal of work, are peculiar to our profession, than which there is none greater, none more beneficent. They are fortified by the dictates of altruism; they are sustained by tradition; they are attested by a long list of worthies whose work of the head, the heart, and the hand in ways innumerable overshadows all other work. I confront you, my friends, with these witnesses whose practice confirms their evidence, making them worthy and acceptable witnesses, who testify of the truth, not in the patent-office, but in the homes of the sick and the injured. They cannot all be wrong; surely their labor has not been in vain; they could not be so mistaken in the work of their lives; nor could those who think and practise otherwise be all right. Think of these high-minded men; weigh their motives; contemplate their work; estimate their success, not in the value of things that perish, but in the meaning of things that cannot be destroyed. The work of their heads was inspired by their hearts,—and is of immortal memory.

You are no longer craftsmen; your labor is not in the field of handicraft. You are no longer tradesmen; you are not engaged in trade. You neither buy nor sell. You work not as the craftsman; not as the tradesman; you work as the surgeon; in fact, you are special surgeons. You labor among those who do the highest kind of hand-work. The position you have achieved is all your own. You are what you are, and where you are, because of your own industry; because you have used your faculties to a good purpose; because your tendency has been to emancipate yourselves from trade and craft. Let me congratulate you on your elevation; on your advancement; on the completeness of your evolution. Step by step you have risen in your work from the rudiments of teeth-extraction until you have reached the environments of a profession such as any man may profess and practise with a just and manly pride. And now I appeal to you,—and let not my appeal be in vain; let it not be as the "listing" wind which you do not respect; let it not be as the idle dream which has no substance; but let it be as to men who have eyes and can see, who have ears and can hear, and as to men who reason and can understand; and, finally, I appeal to you, as men who have worked so nobly and so well with the head and the hand, and who are competent to work with the whole heart,—in the name of science and art; in the name of justice and liberality; in the name of all things noble and true; in the name of the Fathers of Medicine; in the name of that lofty sentiment which interfuses every deed which is great and good; I appeal to you, who have shown so much good sense in the past, to put the crowning touch upon the citadel of your work by refining it with the just, wise, and true spirit of the more perfect altruism.

